# Fall Detection Method for Infrared Videos Based on Spatial-Temporal Graph Convolutional Network

**DOI:** 10.3390/s24144647

**Published:** 2024-07-17

**Authors:** Junkai Yang, Yuqing He, Jingxuan Zhu, Zitao Lv, Weiqi Jin

**Affiliations:** MOE Key Laboratory of Optoelectronic Imaging Technology and System, School of Optics and Photonics, Beijing Institute of Technology, Beijing 100081, China

**Keywords:** fall detection, infrared video, skeleton extraction, spatial-temporal graph convolutional network

## Abstract

The timely detection of falls and alerting medical aid is critical for health monitoring in elderly individuals living alone. This paper mainly focuses on issues such as poor adaptability, privacy infringement, and low recognition accuracy associated with traditional visual sensor-based fall detection. We propose an infrared video-based fall detection method utilizing spatial-temporal graph convolutional networks (ST-GCNs) to address these challenges. Our method used fine-tuned AlphaPose to extract 2D human skeleton sequences from infrared videos. Subsequently, the skeleton data was represented in Cartesian and polar coordinates and processed through a two-stream ST-GCN to recognize fall behaviors promptly. To enhance the network’s recognition capability for fall actions, we improved the adjacency matrix of graph convolutional units and introduced multi-scale temporal graph convolution units. To facilitate practical deployment, we optimized time window and network depth of the ST-GCN, striking a balance between model accuracy and speed. The experimental results on a proprietary infrared human action recognition dataset demonstrated that our proposed algorithm accurately identifies fall behaviors with the highest accuracy of 96%. Moreover, our algorithm performed robustly, identifying falls in both near-infrared and thermal-infrared videos.

## 1. Introduction

With the accelerating aging of the population, the number of elderly individuals living alone is gradually increasing, leading to a growing demand for health and safety monitoring among this demographic [1]. According to the World Health Organization, globally, 646,000 people die each year due to falls, with the inability to receive timely assistance for falls being the main cause of injury or death among elderly individuals living alone [2]. To reduce the incidence of falls and their consequences, researchers have developed a variety of human fall detection systems (HFDS). These systems are capable of automatically detecting when an elderly person falls and promptly notifying caregivers or emergency services [3]. In addition, rehabilitation technologies play a crucial role in helping the elderly regain physical strength, improve balance, and prevent subsequent falls [4]. HFDS hold significant theoretical research significance and practical application value, as they can shorten the response time for assistance and reduce the costs of human caretaking.

Currently, HFDS can be categorized by signal acquisition methods into wearable sensors, vision sensors, and ambient sensors [3]. Wearable systems require users to wear sensors at all times, which may be inconvenient, uncomfortable, or prone to detachment. Ambient systems, such as pressure or sound sensors, are highly susceptible to environmental noise interference. Vision systems typically extract targets from images captured by cameras and derive fall detection results through feature analysis, offering high recognition accuracy, low costs, and continuous operation [5]. However, common visible light cameras have weaker image capture capabilities in complex environments or under low-light conditions, making it difficult to achieve around-the-clock monitoring, and they are also prone to infringing on the privacy of the elderly, which limits their practical application.

Infrared (IR) cameras have gained widespread attention due to their distinctive imaging properties, which enable all-weather and low-interference detection, thereby ensuring reliability across diverse environments. The applications in the near infrared (NIR) and thermal infrared (TIR) spectrums are particularly valued for their distinctive advantages. NIR cameras are relatively cost-effective, while TIR cameras can detect the thermal radiation emitted by objects, providing valuable thermal information. Particularly, the TIR cameras at low resolution can provide information about the human body area without revealing facial features under various lighting conditions, which can effectively protect privacy. Thermal infrared sensors include long-wave infrared (LWIR), mid-wave infrared (MWIR), and short-wave infrared (SWIR), which are applied to different scenarios and needs. LWIR, in particular, offers clear night-time imaging and strong anti-interference capabilities, making it more suitable for the fields of security monitoring and detection identification.

Deep learning algorithms for HFDS using vision sensors mainly include convolutional neural networks (CNNs) and recurrent neural networks (RNNs). CNNs are preferred in this domain due to their more extended development period and mature technology. Variants such as 3D CNNs and graph convolutional networks (GCNs) are commonly used. On the other hand, RNNs are primarily used to process motion features of the human body extracted either from the network or manually. Architectures such as long short-term memory and gate recurrent units are also frequently used for this purpose [5].

Action recognition algorithms based on skeleton sequence extraction and classification have found wide application in the field of fall detection, demonstrating stability across various lighting and background conditions. GCNs have emerged as the optimal method for processing such data structures, offering significant advantages in terms of data volume, structure, and computational speed, particularly for human skeletal data with topological structures [6]. The ST-GCN algorithm based on skeleton sequences has shown exceptional performance among various fall detection methods. It extends GCN to spatial-temporal graphs, enabling simultaneous processing of spatial joint connections and temporal dynamic variations. This algorithm exhibits robustness in dynamic environments and complex backgrounds [7].

Human pose detection methods for extracting skeleton sequences mainly consist of top-down and bottom-up approaches. Among these, the top-down method, exemplified by AlphaPose, operates as a staged detection model [8]. It begins with object detection, identifying humans in the image and labeling each human region with rectangular bounding boxes to eliminate interference from non-human objects. Subsequently, it performs keypoint detection on each human region, achieving exceptionally high accuracy. On the other hand, the bottom-up detection method, represented by OpenPose, initially detects skeletal keypoints and then classifies them based on the affinity between connected joints to determine which person they belong to [9]. Although this method offers rapid detection speed, it is susceptible to interference from other objects in the environment, resulting in lower detection accuracy.

Building upon ST-GCN, Zheng et al. [10] used motion features extracted from 2D skeleton sequences within the ST-GCN framework to identify fall actions. They observed a slight decrease in recognition accuracy compared to 3D poses. The fall accuracy rate for 2D skeleton data was 94.1%. Still, they emphasized the ease of deployment in real environments, achieving a frame rate of 23 frames per second on an Ubuntu system with GPUs. Zheng et al. [11] introduced a lightweight fall detection algorithm and employed the TensorRT acceleration engine to enhance the skeleton extraction AlphaPose model, thereby accelerating the inference speed of pose keypoints. Subsequently, they used an ST-GCN to detect and recognize falls and other actions. The method achieved a consistent detection frame rate of approximately 8.33. The accuracy rate on the UR dataset and the Le2i dataset reached 97.28% and 96.86%, respectively. Liu et al. [12] proposed the CPS-GCN (Cartesian-polar stream graph convolutional network) model based on 2D skeleton sequences. Initially, sequences extracted using OpenPose were augmented with additional 2D skeleton sequences derived from 3D skeletons from NTU. The sequences were then transformed into polar coordinates and separately inputted into GCNs for polar and Cartesian coordinates, resulting in improved performance. The accuracy rate on the NTU RGB-D dataset was 91.78%. Addressing the scarcity of publicly available fall datasets, Keskes et al. [13] applied transfer learning techniques, transferring learned features and weights from the action recognition domain to the fall detection domain. They used datasets such as NTU RGB-D to train the ST-GCN model and achieved 100% accuracy. Although these methods achieved high detection accuracy, their reliance on RGB images and substantial computational demands make them less suitable for applications requiring both privacy and real-time capabilities in elderly fall detection systems. Additionally, they face challenges when processing single-channel and low-resolution IR images, which limits their practical applicability and deployment.

Under low-light conditions, RGB images often struggle to clearly represent human features, while IR images can offer distinct advantages by providing clearer visuals. Yang et al. [14] extracted four fall-sensitive features from low-resolution TIR images and used the K-nearest neighbor algorithm to determine fall occurrences, achieving a 91.25% accuracy rate on a self-built dataset. Chen et al. [15] inferred human height in the real world from TIR images and detected falls based on their variations. Ramanujam et al. [16] used NIR cameras to monitor reflective tape on the bodies of elderly individuals, facilitating night-time fall detection. This method employs an approximate ellipse fitting model to classify binary images into falls or normal activities, achieving a 98.57% accuracy rate on a self-built dataset. Zhou et al. [17] amalgamated consecutive frames of low-resolution TIR images into a consolidated large image, effecting a transformation from three-dimensional video to two-dimensional images. Subsequently, they employed ResNet-18 [18] to recognize fall and fall-like actions such as sitting down and lying down, as well as activities of daily living (ADLs) like standing, sitting, lying, and walking, achieving a recognition accuracy of 99.96%. Yao et al. [19] introduced 3D convolution techniques aimed at recognizing actions like sitting down, lying down, standing up, specific distress poses, and falling, achieving a recognition accuracy of 93.8%. While the aforementioned fall detection methods utilizing IR imagery provide privacy protection, they are constrained to single-wavelength IR images. These methods do not fully satisfy the concurrent demands for accuracy, real-time performance, and generalization within elderly fall detection systems.

To overcome the aforementioned challenges, we introduce a non-intrusive IR video-based human fall detection method designed to maintain privacy. The primary contributions of this paper are as follows:

- We use the AlphaPose skeleton extraction algorithm to process low-resolution IR videos and significantly reduce computational costs by downsampling through keyframe extraction. To enhance recognition accuracy, we employ transfer learning strategies and propose a tailored human keypoints model for fall analysis, ultimately achieving rapid and accurate extraction of skeleton keypoints from the IR videos;
- To combat the challenge of low accuracy in fall detection, we propose an enhanced representation method for skeletal features and introduce an optimized two-stream ST-GCN. This approach incorporates expanded partitioning strategies and multi-scale temporal convolutions to more effectively capture the relationships between human posture and motion patterns, thereby facilitating accurate recognition of fall actions;
- To enhance the capabilities of the fall detection system in privacy protection, system stability, and generalization, the proposed fall detection method is applicable to both NIR and TIR video sources. This method is capable of meeting the demands for 24-h monitoring and privacy protection while exhibiting robustness and generalization.

The remainder of the paper is organized as follows: Section 2 presents the proposed human skeleton extraction network and fall detection algorithm. Experimental results are provided in Section 3. Conclusions are drawn in Section 4.

## 2. Methods

**Figure 1:**
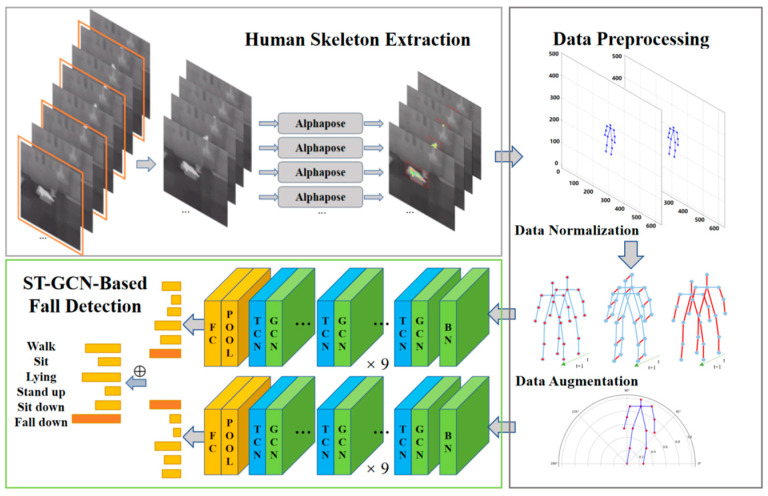
Overall framework of the detection method. Core algorithm ST-GCN consists of BN (batch normalization), GCN (graph convolutional network), TCN (temporal convolutional network), POOL (pooling), and FC (fully connected) layers.

To meet the requirements of precision, real-time capability, generalization, day-and-night monitoring, and privacy protection for elderly fall detection systems, we propose a fall detection model based on ST-GCN utilizing IR video. The overarching architecture, illustrated in Figure 1, consists of a human skeleton extraction module, a data processing module, and an ST-GCN-based fall detection module. The original IR videos are fed into the human skeleton extraction module, where keyframes are extracted and sequentially inputted into the AlphaPose network to obtain human skeleton sequences. The data processing module segments the skeleton sequences into time windows, normalizes the data, generates multi-scale skeleton representations in both Cartesian and polar coordinate systems, and finally feeds the processed skeleton sequences into the ST-GCN-based fall detection module for distinguishing falls and fall-like actions, to ensure the precision and generalization of the model.

### 2.1. Human Skeleton Extraction Methods

**Figure 2:**
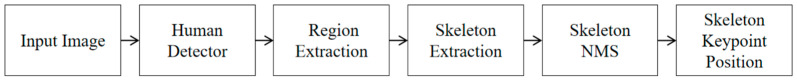
System architecture of AlphaPose.

The lower resolution of IR images and the minimal contrast between the human body and the environment make skeleton keypoints in IR videos susceptible to misidentification. In contrast, the top-down AlphaPose skeleton extraction algorithm initially detects the human target area, thus preventing erroneous connections of human joint positions and enhancing detection accuracy. To swiftly and accurately extract skeleton keypoints from infrared videos, we used a downsampling method by capturing keyframes. Moreover, we introduced a human skeleton model with 13 2D keypoints designed explicitly for fall analysis. Lastly, we enhanced the algorithm’s recognition accuracy through transfer learning strategies. Figure 2 illustrates its pipeline.

The two core modules of AlphaPose are the human detector and the skeleton extration. For the human detector, we used the pre-trained YOLOv4-tiny detector. At the same time, we used FastPose [8] for the pose estimator, which is known for its high accuracy and efficiency. The network architecture primarily consists of ResNet backbone modules and densely connected upsampling convolution (DUC) modules. It employs ResNet as the backbone network to extract features from the input cropped images. Three DUC modules were used to upsample the extracted features, followed by a 1 × 1 convolution layer to generate heat maps. The DUC module first applies 2D convolution to the feature map with dimensions h×c×w and then reshapes it to 2h×2w×c′ through the PixelShuffle operation. FastPose has designed multiple backbone networks for different tasks, including FastRes50 and FastRes152 among others. These backbones have varying detection accuracies and performance.

**Figure 3:**
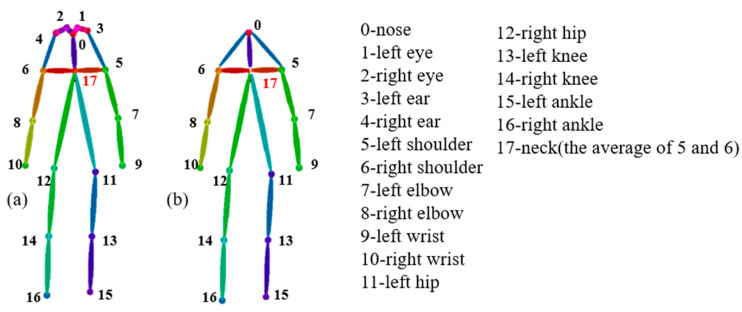
Label order for human body pose keypoints: (**a**) Original 17 2D keypoint model from human keypoint annotation in COCO dataset, (**b**) New 13 2D keypoint model without eyes and ears.

The AlphaPose skeleton extractor demonstrates excellent performance; however, its complex network structure also increases memory consumption. Therefore, we considered extracting keyframes from the original video to shorten the time window of the skeleton data and reduce computational overhead. During video processing, the original video frame rate and the target frame rate were first determined, and the frames were then sampled at intervals based on their ratio. The retrieved keyframes were sequentially inputted into AlphaPose. Initially, the object detection model was used to acquire rectangular bounding boxes for human regions, followed by skeleton extraction on the detected targets, resulting in a detection object list containing keypoints and confidence scores. The human skeletal keypoint model, as illustrated in Figure 3a, depicts the schematic diagram of the original 17 2D keypoints model from the human keypoint annotation in the COCO dataset. This model encompasses key anatomical landmarks such as the eyes, ears, and significant human joints (labeled 0–16), with label 17 denoting the neck, derived as the average of the left and right shoulders. However, for scenarios characterized by large-scale movements like falls, detailed joint information, such as hands and head, proves redundant and has minimal impact on fall recognition accuracy. Hence, we streamlined the sections corresponding to the eyes and ears, simplifying the human anatomical features and reducing the number of model parameters. The resultant keypoint model, as depicted in Figure 3b, comprises 13 2D key points.

**Figure 4:**
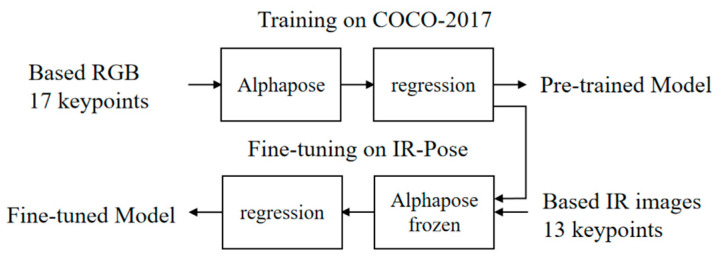
Transfer learning strategy for human skeleton extraction network.

Due to the limited availability of publicly accessible high-quality IR-based fall datasets, the application of transfer learning could harness abundant high-quality annotated data for model pre-training. Subsequently, the acquired generic features were transferred to other models to expedite network parameter convergence and enhance detection performance [20]. Transfer learning typically entails pre-training and fine-tuning models. Initially, a pre-trained model was trained on a large-scale dataset, and subsequently, on a smaller dataset, the parameters of the pre-trained model were employed, with only the final few layers responsible for the classification task being replaced and re-trained. As depicted in Figure 4, the pre-training process involved training on the COCO-2017 RGB pose dataset [21], followed by the adjustment of the network’s fully connected layers. Fine-tuning was then conducted using a self-collected IR pose dataset, facilitating the model’s improved recognition of complex poses such as falls in IR images.

### 2.2. Data Preprocessing

The method based on 2D skeletal keypoint data necessitates considering the influences of viewpoint position, motion scale, global trajectory, and adjacency matrix on the results [22]. The feature information provided by individual keypoint coordinates is quite limited and may not adapt well to different action patterns. Data augmentation to obtain different skeletal feature representations is necessary. Moreover, occlusions in the input human images can lead to the loss of some nodes in the generated skeletal keypoint graphs. In such cases, the feature information regarding human motion encoded in the skeletal keypoint sequence may become inaccurate. Additionally, native human skeletal sequence data may encounter issues such as numerical missing values and a small numerical range during collection. This could result in a temporal jitter in the skeletal sequence, which is irrelevant to the action being performed. Therefore, the normalization of skeletal keypoint coordinates and the completion of missing keypoint coordinates were necessary.

#### 2.2.1. Data Augmentation

**Figure 5:**
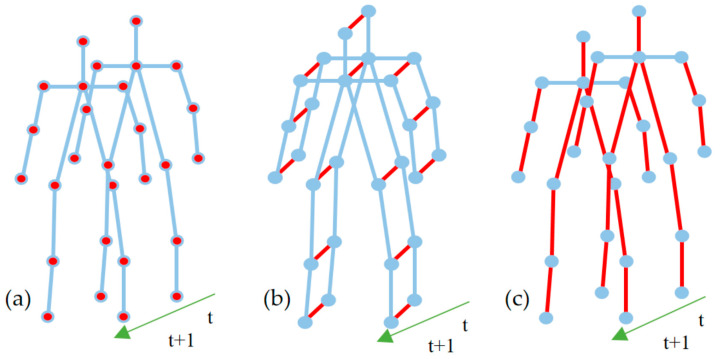
Schematic diagram of multi-feature skeleton data: (**a**) Position features, (**b**) Motion features; (**c**) Bone features. Red represents the features used in the subplot, while blue represents the features that were not used.

In the 2S-AGCN network structure [23], alongside the keypoint position (Positions) features, bone length (Bones) features were also integrated to constitute a two-branch input. This multi-branch input furnishes the network with richer and multi-level feature information. Therefore, to augment the multi-scale information of skeletal data, joint position coordinate information of skeletal keypoints (Figure 5a), motion information in the temporal dimension (Figure 5b), and bone information connecting keypoints in the spatial dimension (Figure 5c) were respectively employed as three branches for feature fusion. Positions are represented as follows:

(1) $$R=\{r_{t,k}|k=0\ldots N,t=1\ldots T\},$$

where $r_{t,k}$ are denoted as (x(t,k),y(t,k)), *k* denotes the index of the keypoint, and *N* represents the maximum index of keypoints. Meanwhile, *t* represents time, ranging from 1 to the maximum length of the skeleton sequence, *T*. 

The motion set $M=\{m_{t,k}|k=0\ldots N,t=1\ldots T\}$ is defined as the difference in coordinates between adjacent frames:

(2) $$m_{t,k}=(x(t+1,k)-x(t,k),y(t+1,k)-y(t,k)),$$

the bone set $B=\{b_{t,k}|k=0\ldots N,t=1\ldots T\}$ is defined as the coordinate differences of specified nodes:

(3) $$b_{t,k}=(x(t,k_{begin})-x(t,k_{end}),y(t,k_{begin})-y(t,k_{end})),$$

here, $\left(k_{begin},k_{end}\right)$ represents the pair of corresponding identifiers for the skeletal joints as shown in Figure 3b.

**Figure 6:**
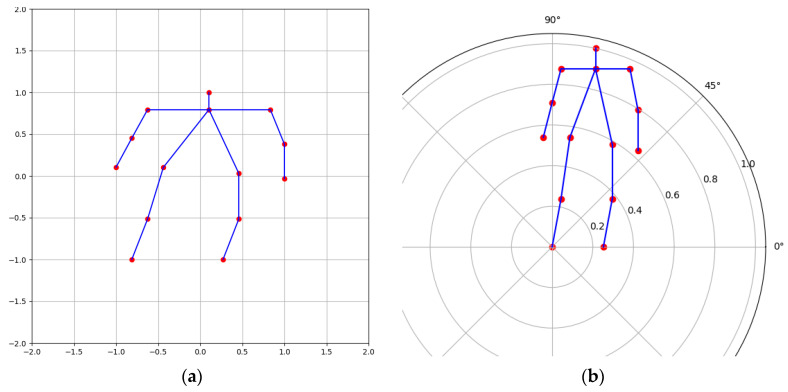
Representation of a 2D skeleton in different coordinate systems: (**a**) Cartesian coordinates, (**b**) Polar coordinates.

To better capture circular movements such as falls, the Cartesian coordinates were transformed into polar coordinates. Polar coordinate representation offers distinct descriptions for position and motion, thereby enhancing and complementing Cartesian coordinates, particularly for specific actions such as falling and waving [8]. As illustrated in Figure 6b, with the right foot (labeled as 10) serving as the polar coordinate origin and the polar axis drawn horizontally to the right, the polar coordinate keypoint set can be expressed as

(4) $$P=\{p_{t,k}|k=0\ldots N,t=1\ldots T\},$$

where $p_{t,k}$ represents polar coordinates $\left(\rho_{t,k},\theta_{t,k}\right)$,

(5) $$\rho_{t,k}=\sqrt{{\left(x_{t,k}-x_{t,10}\right)}^{2}+{\left(y_{t,k}-y_{t,10}\right)}^{2}}$$

(6) $$\theta_{t,k}=\left\{\begin{array}{l} \arctan(\frac{y_{t,10}-y_{t,k}}{x_{t,k}-x_{t,10}}),x_{t,k}=x_{t,10}\\ \pm \pi /2,others \end{array}\right.$$

#### 2.2.2. Data Normalization

The range of 2D skeletal keypoint coordinates extracted by the skeleton extraction network is often excessively large, causing significant variations in human movement. Even minor temporal jitter can profoundly affect the prediction of action categories. Conversely, certain publicly available datasets exhibit keypoint coordinates confined to a narrow range. This limitation leads to densely packed keypoints, posing challenges in distinguishing between action categories effectively. Hence, normalization was applied to scale the range of keypoint coordinates to (−1, 1). The updated coordinates in the Cartesian coordinate system are expressed as:

(7) $$r_{t,k}^{\prime }=\frac{2\times (r_{t,k}-r_{t,k,\min})}{r_{t,k,\max}-r_{t,k,\min}}-1,$$

where $r_{t,k}$ represents the coordinate vector on the x and y components of the *k*th joint at *t* time in a certain action.

The new coordinates in the polar coordinate system are represented as:

(8) $$\rho_{t,k}^{\prime }=\rho_{t,k}/\max(\rho_{t,k}).$$

If certain frames of the skeletal sequence data lacked keypoint information, the missing data could be interpolated by averaging the information from the preceding and subsequent frames. This can be mathematically represented by

(9) $$r_{t,k}=\frac{r_{t-1,k}+r_{t+1,k}}{2}.$$

### 2.3. ST-GCN-Based Fall Detection

**Figure 7:**
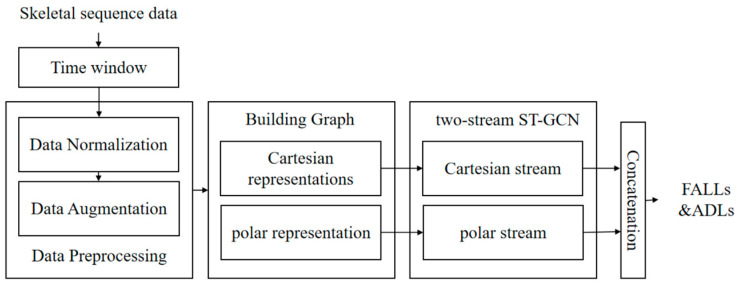
Overall workflow of the fall detection method.

This section delineates the implementation process of the fall detection method based on an ST-GCN, as illustrated in Figure 7. Initially, skeletal sequence data was extracted within a specified time window. Subsequently, data preprocessing was performed to acquire Cartesian and polar representations of the skeleton data. Then, the skeletal graph was constructed and inputted into two identical branches of the ST-GCN for training. Finally, fall detection was conducted using the trained two-stream network. 

#### 2.3.1. Graph and Graph Convolutional Networks

An ST-GCN is a network architecture designed based on graphs, with its input comprising a joint coordinate vector of graph nodes. Therefore, skeletal sequence data must be transformed into spatial-temporal graphs. To integrate joint information along both spatial and temporal dimensions, each node in the graph corresponded to a joint of the human body. The graph comprised two types of edges, spatial edges representing natural connections between joints, and temporal edges connecting the same joint across consecutive frames. Through this construction method, skeletal sequence data within a time window could be transformed into a spatial-temporal graph.

Given a time window of T and N body joints per frame, the graph could be constructed as G=(V,E): where $V=\{v_{tk}|k=0\ldots N,t=1\ldots T\}$ represents the set of nodes, and $v_{tk}$ denotes the graph node corresponding to the joint at time t in the spatial-temporal graph. Each graph node contained the x and y coordinates along with its confidence score. E represents the set of edges, which consists of two subsets:

(10) $$E=\{E_{s};E_{F}\},$$

(11) $$E_{S}=\{v_{tk}v_{tj}|t=1\ldots T,(k,j)\in H\},$$

(12) $$E_{F}=\{v_{tk}v_{(t+1)k}|k=0\ldots N\},$$

where $E_{S}$ represents connections between different joints within the same frame, H denotes connections between all joints of the human body, and $E_{F}$ corresponds to trajectory variations of the same joint across adjacent frames. 

Finally, the spatial-temporal graph G is transformed into a feature vector $F(v_{tk})$, which serves as the input to the ST-GCN.

#### 2.3.2. ST-GCN-Based Fall-Detection Algorithm

Action recognition based on video sequences exhibits temporal dependency, requiring the model algorithm to understand both spatial joint connections and temporal dynamics. Since the emergence of GCN-based methods, research has primarily focused on enhancing ST-GCN units and further refining GCN and TCN units [24]. To better capture spatial features of joint nodes and temporal dependencies, and to accommodate fall action data, optimizations were applied to the spatial-temporal graph convolutional units.

In GCN units, it is crucial to consider how to better extract the topological structure information of the skeleton. At frame t, $E_{S}$ is represented by the adjacency matrix $A\in R^{N\times N}$, where each element $a_{kj}$ reflects the dependency between joints $v_{k}$ and $v_{j}$. 

Graph convolution methods use weights W to aggregate features $v_{k}$ from neighboring vertices of $A\in R^{N\times N}$, updating its feature $f_{i}$,

(13) $$f_{i}=\sum_{v_{j}\in N(v_{k})}a_{kj}x_{j}W.$$

**Figure 8:**
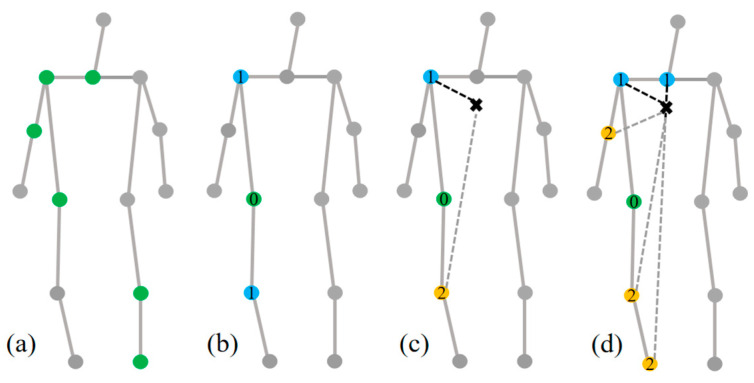
Constructing a partitioning strategy for adjacency matrices: (**a**) Single label, an example where green represents a subset of nodes in a graph. (**b**) Distance, the green nodes represent one category, denoted by the number 0, and the blue nodes represent another category, denoted by the number 1. (**c**) Spatial, taking the green nodes as an example, yellow nodes are added as a third category, denoted by the number 2. (**d**) Expanded spatial, taking the green nodes as an example, the number of new nodes has been added to each category.

The adjacency matrix is critical for extracting the topological structure information of the skeleton, and selecting an appropriate adjacency matrix is crucial for successful skeleton extraction. The original ST-GCN offers three partition strategies for constructing the adjacency matrix, as delineated in Figure 8: (a) single label partitioning strategy, grouping the neighborhood nodes of each node into one subset (including itself); (b) distance partitioning, placing the central node in one class and adjacent nodes (excluding itself) in another; (c) spatial configuration partitioning, dividing the 1-neighborhood of a node into three subsets, the first containing the node itself, the second including neighboring nodes closer to the centroid of the entire skeleton, and the third encompassing neighboring nodes farther from the centroid. 

**Figure 9:**
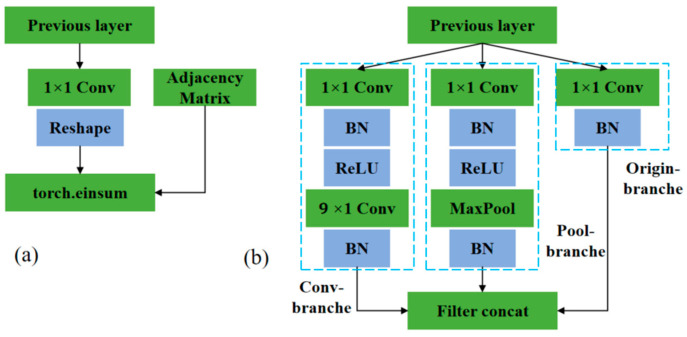
Spatial-temporal graph convolutional unit: (**a**) GCN unit, (**b**) Multi-scale TCN unit.

To better identify fall actions and emphasize the global features of human joints, we proposed a novel spatial configuration partitioning strategy for constructing the spatial graph, as illustrated in Figure 8d. This strategy extends the node neighborhood span to a 2-neighborhood, divided into three subsets: a static group equidistant from the centroid as the root node; a centripetal group composed of nodes closer to the centroid; and a centrifugal group consisting of nodes farther from the centroid. Following the construction of the adjacency matrix using the new partition strategy, it was multiplied with the input skeleton information utilizing the Einstein summation convention tensor to extract spatial features at different scales, as depicted in Figure 9a.

In the TCN unit, to facilitate stronger connections between features across various time scales, a multi-scale TCN module was constructed, drawing upon the framework of GoogLeNet [25], as illustrated in Figure 9b. This module incorporated three fundamental branches denoted as Conv-Branche (Conv-B), Pool-Brabche (Pool-B), and Orgin-Branche (Org-B), interconnected to produce the output. Conv-B predominantly comprised two convolutional layers. Org-B was a basic 1×1 convolutional layer with normalization capabilities. Pool-B was primarily composed of convolutional layers and max-pooling layers, resembling Conv-B in its structure.

A two-stream ST-GCN network was established using the refined spatial-temporal graph convolutional unit. Fusion information encompassed joint position coordinate features, motion features, and bone features in the Cartesian coordinate system within one branch. In contrast, joint position coordinate features in the polar coordinate system comprised the other branch. Each branch was fed into a distinct ST-GCN for feature extraction, with parameters operating independently. Subsequently, these branches of information were concatenated and then classified using a softmax classifier to achieve recognition of falling actions.

## 3. Experiments

### 3.1. Dataset

The experiments used datasets focusing on skeleton extraction and fall detection. Two datasets were employed for skeleton extraction. Initially, the publicly accessible COCO-2017 RGB skeleton extraction dataset served as the pre-training dataset, comprising over 200,000 visible light images, with target and keypoint detection chosen as label types. Considering the inadequate manual annotation of skeletal information in most publicly available IR datasets, particularly for complex actions like falls, we gathered IR videos and extracted images to annotate human bodies, creating the self-collected IR-Pose dataset. The meticulously annotated self-collected IR-Pose dataset was used for fine-tuning purposes. The IR videos contained 2838 samples, including 2696 NIR videos and 142 TIR videos. The NIR videos were captured using a depth camera with a resolution of 512 × 424 pixels and a frame rate of 30 FPS, each lasting 1–2 s. These videos were sourced from NTU-RGB+D-120 [26] datasets. The TIR videos were captured using a HIMICRO HM-TJP10B-3AMF camera with a resolution of 160 × 120 pixels and a frame rate of 9 FPS, each lasting 4–5 s. All videos contained a single person and were recorded in indoor scenes, encompassing both falls and daily activities such as sitting down, standing up, lying, sitting, and walking.

**Figure 10:**
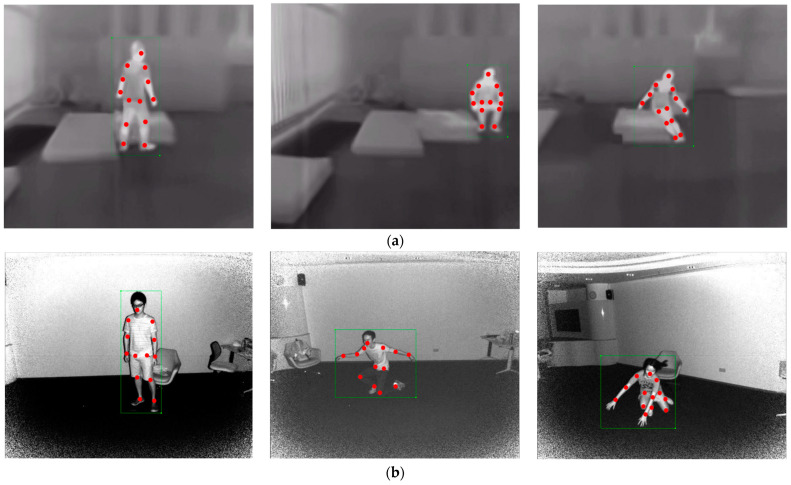
Examples of extracted frames and skeleton keypoints from partial video: (**a**) Thermal infrared data, (**b**) Near infrared data. The dots in the figure represent the key points of the skeleton, and the boxes represent the area of the rectangular regions of the human body.

The IR-Pose dataset originated from keyframe images in both NIR and TIR videos, encompassing the entire process of a person falling. It was used to train a human skeleton extraction model, ensuring that the algorithm could successfully extract skeletal information during operation. Moreover, deliberate efforts were made to incorporate variations in actions, perspectives, and subjects across each image. The data distribution of IR-Pose is outlined in Table 1, with label information comprising 13 keypoints and the human body target, as illustrated in Figure 10.

The skeleton action recognition experiments used two datasets for training and testing, the publicly accessible NTU-RGB+D-120 dataset and the self-compiled IR-Fall dataset. The NTU-RGB+D-120 dataset provided skeleton data for analyzing falls and ADLs. We identified 11 distinct classes of fall-related 3D skeleton data, encompassing a total of 7104 samples. To standardize the training data, the original 25-keypoint 3D skeleton data were converted into 13-keypoint 2D skeleton data. Table 2 provides a breakdown of the dataset composition, segmented into training and testing sets based on different camera viewpoints, referred to as cross-view.

**Figure 11:**
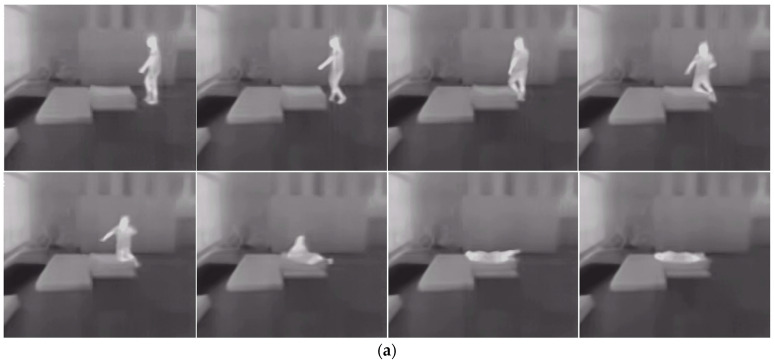

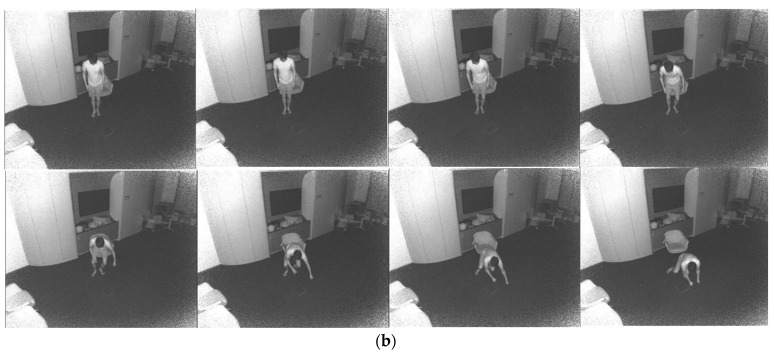
Example of image data contained in a complete action sample from IR-Fall dataset: (**a**) Example of TIR fall data, (**b**) Example of NIR fall data.

The IR-Fall dataset, which integrates both NIR and TIR fall data, was compiled from collected infrared videos and was used to test the algorithm’s capability for cross-spectral fall detection. Distinct actions were isolated, and skeleton data was generated using the AlphaPose method. To mitigate discrepancies in video parameters and thermal imager settings affecting subsequent processing, all video frames were standardized to a resolution of 640×512. Furthermore, a fixed time window of 18 frames was established, with each action typically spanning approximately 2 s. Frames were sampled at a rate of 9 frames per second to ensure complete action samples, as illustrated in Figure 11, and skeleton sequences were derived using AlphaPose. This dataset encompassed six action categories, including “Fall down”, “Sit down”, “Stand up”, and “Walk”, along with two static action categories, “Lying” and “Sit”, totaling 5407 skeleton samples. The dataset was divided into training and testing subsets according to Table 3, with an even distribution of NIR and TIR data.

### 3.2. Experimental Setup

The hardware configuration for this experiment is detailed in Table 4. Throughout the training process, the network model was trained utilizing the cross-entropy loss function. The cross-entropy loss function *L* for multi-task classification can be mathematically represented as follows:

(14) $$L=\frac{1}{N}\sum_{I}L_{i}=-\frac{1}{N}\sum_{i}\sum_{C=1}^{M}y_{ic}\log(p_{ic}),$$

where N represents the number of samples, M represents the number of action categories, $y_{ic}$ represents the sign function, which takes the value of 1 when the true category of sample i equals c, and otherwise 0, and $p_{ic}$ represents the predicted probability that observation sample *i* belongs to category c.

The optimizer adopted the Adam algorithm to optimize the network parameters, with default values of 0.9 and 0.999 for the weighted parameters of the first and second moments, respectively. The learning rate was set to 0.001. A time window of 18 frames was used, with 13 selected keypoints being extracted. The channels were either the Cartesian or polar coordinates for each keypoint. The batch size was set to 32, and the training epochs were set to 30. Therefore, each batch input size was 32, 2, 18, and 14.

### 3.3. Evaluation Criteria

Skeleton extraction commonly employs object keypoint similarity (*OKS*) and average precision (*AP*) as evaluation metrics. *OKS* is a scalar used to compute the similarity between the ground truth and predicted human body keypoints. If the *p*th person’s keypoint is *i* in the image, the *OKS* calculation is as follows:

(15) $$OKS_{p}=\frac{\sum {\;}_{i}\exp\{-d_{pi}^{2}/2S_{p}^{2}\sigma_{i}^{2}\}\delta (v_{pi}>0)}{\sum \delta (v_{pi}>0)},$$

where $d_{pi}$ is the Euclidean distance between the ground truth keypoint and the corresponding predicted keypoint, $S_{p}$ is the scale factor, which is the square root of the pedestrian detection area, δ is the normalization factor (a constant), and $v_{pi}$ represents the visibility of the keypoint. The normalization factor, denoted as $\sigma_{i}$, is a constant for a keypoint of type *i*.

After calculating the similarity for each image, given a threshold T, *AP* was computed through the *OKS* values across all images using the following formula:

(16) $$AP=\frac{\sum {\;}_{p}\delta (OKS_{p}>T)}{\sum {\;}_{p}1},$$

(17) $$mAP=\frac{1}{N}\sum_{i=1}^{N}AP_{i}.$$

The common evaluation metrics for action recognition networks typically include accuracy, precision, recall, and F1 Score. Accuracy is a simple and intuitive evaluation metric, representing the ratio of the number of correctly identified actions to the total number of actions. It provides a clear overall picture of the network model’s performance. Precision and recall are metrics that evaluate individual categories, while the F1 score refers to the harmonic mean of precision and recall. Considering the uneven distribution of NIR and TIR video data samples in the IR-Fall dataset, the F1 score may be biased towards minority classes, as it gives them more significance in imbalanced datasets, which may not accurately reflect the model’s performance on the majority classes. Therefore, we adopted accuracy as the evaluation metric.

Accuracy is measured by:

(18) $$A\mathrm{ccuracy}=\sum_{i=1}^{C}T_{i}/\sum_{i=1}^{C}\left(N_{i}-T_{i}\right),$$

where C represents the total number of samples, $N_{i}$ denotes the total number of samples in the ith class, and $T_{i}$ signifies the number of correctly recognized samples in the ith class.

The number of parameters (Params) in a neural network refers to the total number of parameters contained in the model, which directly determines the model’s size and also affects the memory usage during inference. The calculation equation for a standard convolutional layer is as follows:

(19) $$Params=K_{h}\times K_{w}\times C_{in}\times C_{out},$$

where $K_{h}\times K_{w}$ represents the size of the convolutional kernel, and $C_{in}$ and $C_{out}$ represent the numbers of input and output channels, respectively.

### 3.4. Human Skeleton Extraction Network

First, a pre-trained model was obtained by training on the COCO-2017 dataset. Then, based on the convergence results of the model, the initialization of the network feature extraction part was performed. Finally, fine-tuning was conducted on the accurately annotated infrared pose dataset, IR-Pose. In specific experiments, we considered three different backbone networks within the AlphaPose architecture, aiming to obtain models with high accuracy and low energy consumption, which are used for establishing skeleton labels and practical applications, respectively. ResNet50, composed of residual blocks, is a versatile convolutional neural network model commonly employed for image classification, object detection, and semantic segmentation tasks. This model comprises 50 convolutional layers. FastRes50, a variant of the FastPose model, is optimized based on ResNet50 to meet the requirements of skeleton extraction tasks. FastRes152, on the other hand, is a further optimization of FastRes50 with deeper network layers capable of capturing richer and more complex features. However, due to the increased number of parameters, FastRes152 demands more computational resources and memory support. 

The comparative results of their performance are shown in Table 5. We can see AlphaPose (FastRes152) demonstrates superior performance in the mAP metric, rendering it suitable for generating skeleton labels. Conversely, AlphaPose (FastRes50) excels in FPS and FLOPS metrics, making it well-suited for real-time inference tasks.

Table 6 compares the disparities in skeleton extraction models across two distinct types of data. In contrast to TIR datasets, NIR datasets exhibit higher image resolution, accentuating the texture features of the human body and aligning more closely with the features observed in visible light images. Upon scrutinizing three distinct AlphaPose models, it was discerned that all models demonstrated superior performance on the NIR dataset. This tendency could be attributed to the NIR dataset providing more detailed information, which allows skeleton extraction models to identify the key keypoints of the human body with higher precision. In this context, the higher resolution and more distinct texture features of the NIR dataset may facilitate the model’s ability to learn and infer poses, consequently improving overall performance. Consequently, for skeleton extraction tasks, NIR datasets might hold an advantage over TIR datasets, especially when it comes to capturing fine human texture details.

**Figure 12:**
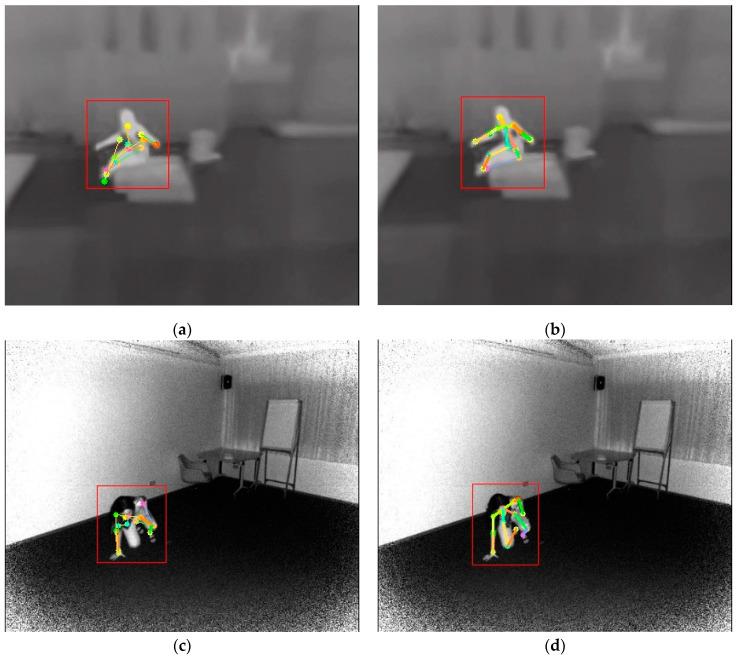
Comparison of test results of pre-trained and fine-tuned models: (**a**) NIR image test results using pre-trained model, (**b**) NIR image test results using fine-tuned model, (**c**) TIR image test results using pre-trained model, (**d**) TIR image test results using fine-tuned model.

Based on the convergence results of the aforementioned skeleton extraction models, the backbone network with the lowest FLOPS indicator was selected. Visual analysis was conducted on falling actions in the infrared videos using AlphaPose (FastRes50), as shown in Figure 12. The pre-trained model trained on the RGB dataset performed well in predicting standing poses but poorly in predicting falling poses. The fine-tuned model inherited the ability of the pre-trained model to predict standing poses, and after adapting to the IR images and special actions, it significantly improved the prediction accuracy of falling.

### 3.5. ST-GCN-Based Fall-Detection Network

To thoroughly validate the optimization methods proposed in this paper, we conducted ablation experiments using the NTU-RGB+D-120 and IR-Fall datasets, evaluating multiple feature skeleton data, expanded spatial strategies, and multi-scale temporal convolutions.

#### 3.5.1. Effects of Different Skeleton Data Representations on ST-GCN Performance

Table 7 presents the impact of different forms of skeleton data on detection accuracy. Our proposed two-stream model performed effectively on both datasets, with the two-stream model incorporating polar coordinates and joint representation, achieving the highest performance in the IR-Fall dataset. Conversely, in the NTU-RGB+D-120 dataset, the two-stream model with motion and joint representation outperformed the others. To assess the significance of each skeletal feature representation in detection accuracy, we conducted comparative experiments with one-stream configurations. In the one-stream structure, polar coordinate representation demonstrated higher accuracy on both datasets, proving its effectiveness. However, significant differences in the performance of motion representation were observed, with the best performance on the NTU-RGB+D-120 dataset but significantly poorer performance on the IR-Fall dataset, achieving only 67.23% accuracy. This discrepancy arose from the model’s difficulty in distinguishing static action categories such as “lying” and “sit”, where the changes between frames were minimal or negligible. Motion features, designed to differentiate actions based on differences between consecutive frames, proved unsuitable for distinguishing falls and similar actions.

**Figure 13:**
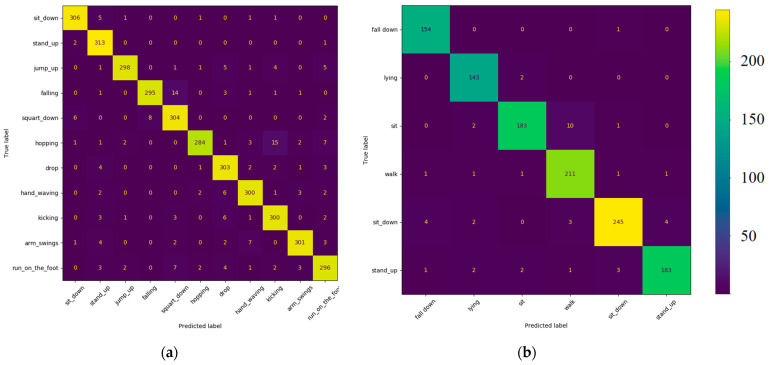
Confusion matrix comparison of two-stream model on two datasets: (**a**) Results on NTU-RGB+D-120 dataset; (**b**) Results on IR-fall dataset.

To observe the model’s recognition performance for each action category, we conducted a confusion matrix analysis. Figure 13 depicts the performance of the two-stream model on the two datasets, where the vertical axis denotes the true labels, the horizontal axis signifies the predicted labels, and the color intensity reflects the number of predicted samples. Ideally, a diagonal line should be evident. Figure 13a illustrates the confusion matrix results on the NTU-RGB+D-120 dataset, showcasing a generally commendable overall recognition performance of the model. Nonetheless, some misclassifications were observed, particularly in falls and squatting down. Similarly, Figure 13b displays the confusion matrix results on the IR-fall dataset, indicating the model’s outstanding overall recognition performance. Specifically, the recognition accuracy for fall actions was high. However, misclassifications between sit and walk actions were observed.

#### 3.5.2. Effects of GCN and TCN on ST-GCN Performance

Table 8 presents the results of evaluating the influence of different partitioning strategies on detection accuracy. The experimental results indicate that the expanded spatial strategy consistently achieves the best performance across all experiments. This suggests that employing a novel spatial configuration partitioning strategy aids in better capturing the global features of human joints when constructing adjacency matrices, making the model more sensitive to global motions.

Table 9 presents the experimental results testing the influence of different scales of TCN on detection accuracy compared to the baseline. The baseline consisted of the original TCN unit in the ST-GCN, primarily composed of a 9 × 1 convolutional block. The multi-scale TCN aggregates were featured at different temporal scales. Models incorporating branches of Conv-B, Pool-B, and Org-B multi-scale configurations outperformed the baseline model in terms of detection accuracy. This result indicates that using multi-scale TCN can better capture key features at different temporal scales, thereby improving action detection accuracy. Employing multi-scale models allows for a more comprehensive consideration of temporal information changes during the action process, making the model more flexible and robust in analyzing different actions.

#### 3.5.3. Effects of Network Layers and Temporal Windows on ST-GCN Performance

The network’s depth and width significantly impacted the model’s accuracy and inference speed. The original ST-GCN model consisted of a 10-layer network structure. Expanding on this foundation, we performed pruning experiments that concurrently reduced the number of channels and layers within the network. The experimental results indicate that as the network depth decreases, there is no significant loss in the model’s accuracy across both datasets. Indeed, accuracy improved in some instances with a reduced number of layers. This outcome could be attributed to the relatively straightforward action patterns and the limited data volume present in both datasets.

Furthermore, excessively complex network structures may result in overfitting. Therefore, the judicious selection of network depth and width is pivotal to model performance, underscoring the importance of a deliberate design tailored to the dataset’s traits and the task’s demands. In the pruning experiments of Table 10, the 9-layer network configuration demonstrated the highest recognition accuracy on both datasets, making it the most optimal choice regarding the number of layers. 

Concerning the ST-GCN, the choice of time window significantly influenced detection results. Table 11 explores the ramifications of different time windows and assesses their efficacy on two datasets. The 12-frame and 15-frame windows, directly derived from the 18-frame window, exhibited reduced accuracy due to the absence of crucial frame information. Conversely, the 9-frame window, obtained by selecting frames at intervals from the 18-frame window, retained action integrity but lacked some temporal dimension information, also resulting in diminished accuracy. Given the 9 FPS frame rate of the TIR videos in the dataset, each action could be captured for a maximum of 18 frames within a specified time period. Consequently, we conducted tests with 24-frame and 30-frame windows on the NTU-RGB-D-120 dataset. Although there was a marginal enhancement in detection accuracy, the overall algorithm frame rate significantly decreased. This decline was particularly evident with a 30-frame window, as the algorithm was unable to complete the time window with skeleton data within the 2-s action duration. This limitation renders the approach unsuitable for practical applications.

The experimental results underscore the need to strike a balance between the comprehensiveness of keyframes and the sufficiency of temporal dimension information when selecting a time window. A concise window may result in incomplete information, while an overly long window could introduce redundant data or overlook essential action details. Therefore, in practical applications, selecting an appropriate time window size requires thoughtful consideration of task specifics and dataset characteristics to enhance detection performance. Our ultimate suggestion is to adopt an 18-frame time window, as it meets the real-time performance and accuracy requirements for HFDS.

#### 3.5.4. Effects of Different Infrared Datasets on ST-GCN Performance

Table 12 evaluates the performance of our skeleton action recognition model when trained on two distinct data types, revealing superior performance on the NIR dataset compared to the TIR data across various feature inputs. We attributed this observation to two potential factors: (1) the limited capability of the skeleton extraction model to accurately extract poses from the TIR images, resulting in imprecise prediction of skeleton keypoints, and (2) the optimal performance on the TIR dataset may be constrained due to the limited data volume. Nonetheless, the two-stream results, employing both polar coordinates and skeleton representation, consistently achieved the highest performance across disparate datasets. This underscores the efficacy of our model in extracting core features pertinent to falling and fall-like actions, thereby facilitating their discrimination and recognition.

Table 13 evaluates the performance of the models trained on a unified dataset encompassing both IR datasets for each action type. It reveals that the models trained on the combined dataset achieved higher recognition accuracy than those trained on each dataset separately, as shown in Table 13. This finding implies that standardizing the data type and time window during data preprocessing allows samples from different datasets to complement one another. Notably, for the two primary action categories central to falling behavior in both datasets, namely “Fall down” and “Lying”, the recognition accuracy was high. This high accuracy signifies our model’s heightened sensitivity to falling actions and its adept recognition ability for other actions, thereby showcasing its versatility across various daily actions. These experimental findings affirm that our devised model successfully meets its intended objective. It proficiently uncovers and extracts key features amidst diverse action patterns, accurately discerning falling actions with precision, real-time performance, and generalization capability.

#### 3.5.5. Comparison with Other Fall Detection Methods

Table 14 presents a comparative analysis between the proposed method and other approaches, demonstrating the highest accuracy on the self-constructed IR-Fall dataset. In comparison to other GCN-based methods, this study optimized the spatial-temporal graph convolutional units and employed a two-stream structure utilizing Cartesian and polar coordinate systems, leading to a significant enhancement in fall-action recognition accuracy. Moreover, pruning of the spatial-temporal graph convolutional units was executed, resulting in a noteworthy reduction in model parameters and computational expenses.

The ablation experiments conducted on the NTU-RGB+D-120 and IR-Fall datasets have rigorously validated the efficacy of the optimization techniques proposed in this study for action recognition. By systematically evaluating various feature skeleton data, expanding partitioning strategies, and incorporating multi-scale temporal convolutions, we have successfully optimized the model structure and parameters. Our approach demonstrates outstanding performance, particularly in fall-action recognition tasks.

## 4. Conclusions

This study introduces a fall detection model based on an ST-GCN for IR videos, aiming to address the limitations associated with traditional vision sensor-based methods. These limitations include poor adaptability, privacy concerns, and low recognition accuracy. The model leverages low-resolution IR videos, maintaining robustness against external environmental factors while safeguarding user privacy. It also demonstrates versatility by accommodating both NIR and TIR image inputs. We designed an infrared video skeleton extraction algorithm based on the AlphaPose model. The method first detects all human rectangular areas in the image and then performs skeleton extraction and tracking for each area. By adjusting the network structure and employing transfer learning strategies to fine-tune the model, we enhanced the algorithm’s ability to extract skeletons from infrared videos. Additionally, we significantly reduced computational costs through keyframe extraction for video downsampling. The algorithm is applicable to both near-infrared and thermal infrared images, capable of rapidly and accurately extracting human skeleton sequences from infrared videos. We also proposed a dual-stream spatio-temporal graph convolutional fall detection algorithm based on skeleton sequences. By combining the polar coordinate representation and joint representation of the skeleton sequences in a dual-branch feature, we enhanced the model’s generalization capability. Using an adjacency matrix constructed with expanded spatial strategies and multi-scale temporal graph convolutional units, we more effectively captured the relationships between human posture and motion patterns, thereby enhancing the model’s ability to effectively recognize fall actions. Furthermore, by pruning network layers to reduce the number of model parameters, we improved the model’s real-time responsiveness. The experimental results show that the proposed infrared fall detection model achieves high accuracy on both public and self-collected datasets, meeting the requirements for real-time fall detection. The final algorithm can meet the system’s requirements for detection accuracy and real-time response, and is suitable for single- or multiple-person scenarios in both NIR and TIR videos.

Future research could explore the integration of self-attention mechanisms to enhance fall detection performance. This approach would consider the potential topological relationships between human joints based on actual motion characteristics.

## Figures and Tables

**Table 1:** Classification of IR-Pose image datasets.

Category	Image Resolution	Train	Test
Near infrared	512 × 424	720	160
Thermal infrared	160 × 120	320	60

**Table 2:** Classification of skeleton sequence samples for different actions in NTU-RGB+D-120 datasets.

Category	Description	Train	Test
FALL	Fall down	630	316
ADLs	Stand up	620	316
	Sit down	626	315
	Squat down	638	320
	Jump up	632	316
	Hopping	631	316
	Drop	626	316
	Hand waving	628	316
	Kicking something	630	316
	Arm swings	640	320
	Run on the foot	640	320

**Table 3:** Classification of skeleton sequence samples for different actions in IR-Fall datasets.

Category	Description	Train		Test	
		TIR	NIR	TIR	NIR
FALL	Fall down	600	105	130	25
ADLs	Sit down	600	77	240	18
	Stand up	650	84	170	22
	Lying	586	82	129	16
	Sit	747	67	176	20
	Walk	518	199	180	36

**Table 4:** Hardware configuration.

Name	Configuration Information
Processor	Intel Xeon 4110*2 (from Intel Corporation in Santa Clara, CA, USA)
Memory	32.0 GB
Operating system	Centos7
GPU support	Nvidia Gv100, CUDA 9.0, cuDNN 7.0

**Table 5:** Performance comparison of AlphaPose models on IR-Pose.

Branch	Params/MB	FPS	FLOPS/G	mAP
AlphaPose (FastRes50)	38.70	33.79	20.87	0.77
AlphaPose (Res50)	34.00	33.17	34.16	0.83
AlphaPose (FastRes152)	79.30	19.59	56.43	0.87

**Table 6:** Performance comparison of AlphaPose models on IR-Pose dataset.

Branch	NIR	TIR
AlphaPose (FastRes50)	0.8317	0.7173
AlphaPose (Res50)	0.8525	0.7469
AlphaPose (FastRes152)	0.8776	0.7649

Branch

NIR

TIR

**Table 7:** Accuracy comparison for different forms of skeleton data on two datasets.

Coordinates	Features	NTU-RGB+D-120	IR-Fall
One-Stream (Multi-scale TCN unit/ Expanded spatial)	Positions	0.8996	0.9369
	Motions	0.9182	0.6723
	Bones	0.9081	0.9595
	Polar	0.9168	0.9535
Two-Stream (Multi-scale TCN unit/ Expanded spatial)	Positions & Motions	0.9464	0.9468
	Positions & Bones	0.9085	0.9502
	Motions & Bones	0.9268	0.9476
	Polar & Positions	0.9217	0.9552
	Polar & Motions	0.9188	0.9527
	Polar & Bones	0.9257	0.9637

**Table 8:** Accuracy comparison for different partitioning strategies on two datasets.

Strategies	NTU-RGB+D-120	IR-Fall
Single label	0.9271	0.9257
Distance	0.9289	0.9527
Spatial	0.9312	0.9556
Expanded spatial	0.9464	0.9637

**Table 9:** Accuracy comparison for different scales of TCN on two datasets.

Branch	NTU-RGB+D-120	IR-Fall
Baseline	0.9134	0.9468
Conv-B	0.9409	0.9510
Conv-B & Pool-B	0.9369	0.9519
Conv-B & Org-B	0.9320	0.9578
Conv-B & Pool-B & Org-B	0.9464	0.9637

**Table 10:** Performance comparison of ST-GCN layer on two datasets.

	NTU-RGB-D-120	IR-Fall	Params/MB
Layer = 10, depth = 256	0.9340	0.9502	2.410
Layer = 9, depth = 256	0.9464	0.9637	1.766
Layer = 8, depth = 256	0.9335	0.9586	1.121
Layer = 7, depth = 128	0.9395	0.9527	0.5987
Layer = 6, depth = 128	0.9266	0.9525	0.5577
Layer = 5, depth = 128	0.9200	0.9578	0.2745
Layer = 4, depth = 64	0.9332	0.9611	0.1422
Layer = 3, depth = 64	0.9180	0.9535	0.1012
Layer = 2, depth = 64	0.9005	0.9459	0.0602

**Table 11:** Impact of different time windows on accuracy.

Frame Window	Accuracy of ST-GCN		Speed of the Entire Algorithm (FPS)
	NTU-RGB-D-120	IR-Fall	
9	0.8535	0.9358	35.3
12	0.9289	0.9181	32.3
15	0.9349	0.9527	29.9
18	0.9464	0.9637	25.5
24	0.9498		18.8
30	0.9552		11.2

**Table 12:** Accuracy comparison for different forms of skeleton data on IR-Fall dataset.

Coordinates	Features	NIR	TIR
One-Stream (Multi-scale TCN unit/ Expanded spatial)	Positions	0.9431	0.8812
	Motions	0.6219	0.7188
	Bones	0.9403	0.8125
	Polar	0.9521	0.8875
Two-Stream (Multi-scale TCN unit/ Expanded spatial)	Positions & Motions	0.9343	0.8313
	Positions & Bones	0.9421	0.7875
	Motions & Bones	0.9510	0.8313
	Polar & Positions	0.9609	0.8625
	Polar & Motions	0.9589	0.8375
	Polar & Bones	0.9628	0.9187

**Table 13:** Accuracy comparison for different actions on IR-Fall dataset.

Model Structure	Features	NIR	TIR
Polar & Bones/ Multi-scale TCN unit/ Expanded spatial	Fall down	0.9845	1.0000
	Lying	0.9845	1.0000
	Sit	0.9489	0.8500
	Walk	0.9333	0.9167
	Sit down	0.9536	0.9524
	Stand up	0.9826	0.9534

**Table 14:** Performance comparison among fall detection approaches on two datasets.

Methods	Accuracy		Params/MB	FLOPS/G
	NTU-RGB+D-120	IR-Fall	3.067	0.29
ST-GCN [7]	0.9048	0.9500	6.132	0.58
CPS-GCN [10]	0.9246	0.9535	3.278	0.33
AS-GCN [27]	0.9186	0.9467	1.766	0.21
Our method	0.9464	0.9637	3.067	0.29

## Data Availability

Data are contained within the article.
